# Lower reference limits of quantitative cord glucose-6-phosphate dehydrogenase estimated from healthy term neonates according to the clinical and laboratory standards institute guidelines: a cross sectional retrospective study

**DOI:** 10.1186/1471-2431-13-137

**Published:** 2013-09-10

**Authors:** Sameer Yaseen Al-Abdi, Amina Suleman Alsaigh, Fahima Lugman Aldawoud, Amal Ali Al Sadiq

**Affiliations:** 1 Department of Pediatrics, King Abdulaziz Hospital, Al-Ahsa, Saudi Arabia; 2 Department of Nursing, King Abdulaziz Hospital, PO Box 2477, Al-Ahsa 31982, Saudi Arabia; 3 King Abdullah International Medical Research Center, Riyadh, Saudi Arabia

**Keywords:** Glucose-6-phosphate dehydrogenase, Reference interval, Lower reference limit, Cord blood, Term neonate

## Abstract

**Background:**

Previous studies have reported the lower reference limit (LRL) of quantitative cord glucose-6-phosphate dehydrogenase (G6PD), but they have not used approved international statistical methodology. Using common standards is expecting to yield more true findings. Therefore, we aimed to estimate LRL of quantitative G6PD detection in healthy term neonates by using statistical analyses endorsed by the International Federation of Clinical Chemistry (IFCC) and the Clinical and Laboratory Standards Institute (CLSI) for reference interval estimation.

**Methods:**

This cross sectional retrospective study was performed at King Abdulaziz Hospital, Saudi Arabia, between March 2010 and June 2012. The study monitored consecutive neonates born to mothers from one Arab Muslim tribe that was assumed to have a low prevalence of G6PD-deficiency. Neonates that satisfied the following criteria were included: full-term birth (37 weeks); no admission to the special care nursery; no phototherapy treatment; negative direct antiglobulin test; and fathers of female neonates were from the same mothers’ tribe. The G6PD activity (Units/gram Hemoglobin) was measured spectrophotometrically by an automated kit. This study used statistical analyses endorsed by IFCC and CLSI for reference interval estimation. The 2.5th percentiles and the corresponding 95% confidence intervals (CI) were estimated as LRLs, both in presence and absence of outliers.

**Results:**

207 males and 188 females term neonates who had cord blood quantitative G6PD testing met the inclusion criteria. Method of Horn detected 20 G6PD values as outliers (8 males and 12 females). Distributions of quantitative cord G6PD values exhibited a normal distribution in absence of the outliers only. The Harris-Boyd method and proportion criteria revealed that combined gender LRLs were reliable. The combined bootstrap LRL in presence of the outliers was 10.0 (95% CI: 7.5-10.7) and the combined parametric LRL in absence of the outliers was 11.0 (95% CI: 10.5-11.3).

**Conclusion:**

These results contribute to the LRL of quantitative cord G6PD detection in full-term neonates. They are transferable to another laboratory when pre-analytical factors and testing methods are comparable and the IFCC-CLSI requirements of transference are satisfied. We are suggesting using estimated LRL in absence of the outliers as mislabeling G6PD-deficient neonates as normal is intolerable whereas mislabeling G6PD-normal neonates as deficient is tolerable.

## Background

Glucose-6-phosphate dehydrogenase (G6PD) deficiency is a common X-linked recessive enzymopathy [1]. Genotypically, males are either hemizygous for the G6PD gene with normal gene expression or G6PD deficient, while females can be normal, heterozygous, or homozygous. While about 186 variants have been recognized, not all of them are clinically significant [2]. The World Health Organization (WHO) has classified G6PD variants into five classes: Class I (severe enzyme deficiency with chronic non-spherocytic hemolytic anemia); Class II (<10% of normal); Class III (10–60% of normal); Class IV (60–100% of normal); and Class V (>200% of normal) [3]. The G6PD deficiency is well-known to cause hyperbilirubinemia that may be severe enough to cause kernicterus or neonatal death [1,4]. Early recognition of G6PD deficiency can help prevent these serious complications. Thus, the WHO has endorsed screening cord blood samples from all neonates in populations with a prevalence of G6PD deficiency of 3 to 5% or more in males [5]. The WHO has endorsed 5 methods as screening or diagnostic tests for G6PD deficiency including quantitative measurement of G6PD activity in red blood cells (RBCs) [5,6]. Reference limits are the cornerstones of interpretation of any laboratory result, including quantitative cord G6PD values. Previous studies have reported on the lower reference limit (LRL) or lower decision limit (LDL) of quantitative cord G6PD values, but these have methodological and/or statistical flaws [7-18]. These studies did not implement standard statistics used to estimate LRLs that have long been endorsed by the International Federation of Clinical Chemistry (IFCC) and the Clinical and Laboratory Standards Institute (CLSI) [19-25]. None of these studies have addressed the detection and handling of outliers. Only two of these studies addressed the type of distribution of G6PD values and were in accordance with known methods of defining reference intervals (RIs), including the 95% central RIs [7,8]. Instead of reporting non-parametric RIs that do not make any assumptions about the data distribution, [26] some researchers have based the LRL on the mean without addressing normal distribution testing [13]. Some reported LRLs/LDLs have been estimated from small sample sizes, [7,11,12] from populations with high G6PD deficiency prevalence, [8-10,13,15,16] and from mixtures of G6PD-normal and G6PD-deficient preterm and full-term neonates [8,15]. Therefore, we aimed to estimate the LRLs of quantitative cord G6PD activity from a large population of healthy term neonates in accordance with the standard method of IFCC-CLSI [19-25]. In our opinion, using of these common standards would overcome limitations of the previous studies and yield true findings [27]. We elected not to estimate the upper reference limit as it has no clinical implication [28].

## Methods

### Setting

We conducted this cross sectional retrospective study at King Abdulaziz Hospital (KAH) in the Al-Ahsa area of the Eastern province of Saudi Arabia. Since 2009, KAH has been accredited by the Joint Commission of International Accreditation and its laboratory has been accredited by the College of American Pathologists, which implements the CLSI standards in its accreditation checklists.

### Analytic method for G6PD

In early 2008, KAH began universal cord blood screening for G6PD deficiency coupled with direct antiglobulin testing and blood grouping [29]. A semi-qualitative fluorescence spot test (FST) with a cut-off point of 2.1 Units/gram Hemoglobin (U/g Hb) was used until 01 March 2010, at which point it was replaced with an automated commercial kit. This kit is Udilipse Auto Analyzer from United Diagnostics Industry, Dammam, Saudi Arabia that offers quantitative measurement of G6PD activity.

Just after delivery of the placenta, whole cord blood was collected in ethylenediaminetetraacetic acid (EDTA) Vacutainer tubes (Becton-Dickinson, Rutherford, NJ, USA). Blood specimens were transported to the laboratory by pneumatic tubes. Quantitative G6PD activity measurements were performed in batches every morning, 7 days a week, with blood samples stored in the vertical position at 2–8 degree Celsius (°C) until analysis. The principal method of the Udilipse kit is in accordance with the standardized WHO method for G6PD assay of the hemolysate at 25°C outlined as follows [6]:

$$\begin{array}{c} \mathrm{Glucose}‒6‒\mathrm{Phosphate}+\mathrm{NADP}\;\overset{G6\mathrm{PD}}{\to }6-\text{Phosphogluconate}\\ \;+\mathrm{NADPH}+H^{+} \end{array}$$

The reagents of this kit are outlined as follows:

Reagent 1 (G6PD Buffer): Ready to use 50 micromolar (mM)/Liter triethanolamine buffer, 5 mM/Liter EDTA, pH 7.6 ± 0.05 (25°C).

Reagent 2 (G6PD NADP): Reconstituted 30 mM nicotinamide adenine dinucleotide phosphate (NADP).

Reagent 3 (G6PD Substrate): Reconstituted 17 mM Glucose-6-Phosphate Sodium.

Reagent 4 (G6PD Lysis): Ready to use 0.2% Saponin aqueous solution.

Hemolysate was prepared according to the manufacturer’s manual for the Udilipse kit by adding a well-mixed 100 microliters of whole blood to 400 microliters of 0.2% Saponin. The G6PD activity was measured within one hour of hemolysate preparation. The activity of the G6PD enzyme is measured by the rate of NADPH formation, which is measured spectrophotometrically by means of the increase in extinction at 340, 334 or 365 nanometer. As the Udilipse kit only measures G6PD activity, hemoglobin was measured spectrophotometrically on the same sample by CELL-DYN Sapphire (Abbott Diagnostics, Santa Clara, CA, USA). Then, the values of G6PD activity and hemoglobin were entered and stored in Cerner Lab Information System Software (Cerner Corporation, Kansas City, MO, USA), which is already programmed to express the G6PD activity in U/g Hb by dividing the obtained G6PD activity by the obtained hemoglobin values.

### Reference sample group

The IFCC-CLSI recommends estimating RLs from a healthy population [20,25]. In our case, the healthy population would be the population free from G6PD mutations. The Al-Ahsa area is composed of an oasis part inhabited by Arab Muslims of urban descent and a desert part inhabited by Arab Muslims of Bedouin descent. The overall prevalence of G6PD deficiency in the Al-Ahsa area is 23% in males and 13% in females [30]. The G6PD-Mediterranean (WHO class II) constitutes 84% of G6PD mutations in this population and the G6PD-A^-^ (WHO class III) represents 5.8% [2,28,31]. G6PD deficiency is confined to the oasis part of the area, as malaria was much more prevalent in the oasis than in the desert [32-34]. A recent retrospective Ahsai study found no single severe G6PD deficient case among 236 neonates from one Arabic Bedouin tribe subjected to cord blood screening by FST [29]. Therefore, this tribe can function as the reference population for estimating the LRLs of G6PD [35]. Henceforth, this tribe will be referred to as the reference tribe.

The reference sample group was identified from the delivery room log book via the mothers’ names. It consisted of all consecutive neonates born to mothers from the reference tribe between March, 2010 and June, 2012 that satisfied all the following posteriori inclusion criteria: 1) full-term (37 weeks of gestation); 2) roomed in with mothers and were not admitted to the special care nursery; 3) no phototherapy treatment during the neonatal period; 4) negative direct antiglobulin test; and 5) fathers of female neonates were from the reference tribe. For male neonates it does not matter whether the father is from the reference tribe or not, but it does matter for female neonates as the G6PD is inherited as X-linked recessive. Thus, only copies of birth notices of female neonates were reviewed to ascertain that both parents were from the reference tribe. At KAH, birth notices that include the full name of both parents are issued upon home discharges and copies of these birth notices are stored in hard and electronic medical records of neonates. We selected only full-term neonates as we did not expect to have an adequate number of preterm neonates of the reference sample group during the study period and as G6PD values have been shown to be higher in preterm neonates <34 weeks of gestation [15,36,37]. Gestational age was calculated according to the best obstetric estimate at KAH based on the first or second trimester obstetric ultrasound and/or the last menstrual period, and on the Ballard score when the best obstetric estimate was uncertain [38,39]. The phototherapy threshold used in this hospital has been published previously and is more conservative than those used by the American Academy of Pediatrics [29]. All the study data were extracted from electronic health care records (QuadraMed CPR 5.0.9, Reston, VA, USA). This study was exempted from review by the Institutional Review Board.

### Statistical analysis

We performed statistical analyses as endorsed by the IFCC- CLSI [24,25]. The IFCC-CLSI has endorsed the use of the 2.5th percentile of the values as the LRL. Further, this body endorses the use of a minimum of 146 reference individuals in each gender partition to calculate the 95% confidence interval (CI) of the 2.5th percentile [25]. We estimated the 2.5th percentiles for males, females, and the combined group.

The IFCC-CLSI suggests detecting outliers by Tukey’s boxplot for unskewed data and by method of Horn for skewed data. The method of horn uses Tukey’s boxplot on Box-Cox transformed data [40]. Although the method of Horn is implanted in RefVal 4.11, [41] we also estimated the best lambda and performed the Box-Cox transformation by using already available SPSS syntax [42]. The two Tukey’s inner fences are the 25th percentile minus 1.5 interquartile range (IQR) and the 75th percentile plus 1.5 IQR. The two Tukey’s outer fences are the 25th percentile minus 3.0 IQR and the 75th percentile plus 3.0 IQR. Values outside the inner fences but inside the outer fences are considered mild outliers and values outside the outer fences are considered extreme outliers.

Data extraction and entry of the outliers were double-checked. It was difficult to ascertain whether these outliers were due to G6PD mutations or to pre-analytic errors as these values were not cross-referenced with molecular testing. Assuming that the reference tribe is in Hardy-Weinberg equilibrium, the Hardy-Weinberg equation was used as a surrogate to determine whether these outliers represent G6PD mutations [43,44]. The asymptotic Pearson’s chi-square goodness-of-fit test with 2 degrees of freedom was calculated to test the departure from Hardy-Weinberg proportions [45]. Distributions of G6PD values with and without outliers were presented as histograms with superimposed best-fitting normal distribution curves. Non-integer G6PD values were rounded down to the nearest integer at the boundaries of histogram bins. The type of distribution of the G6PD values was assessed by visual examination of the histograms, comparison between measures of central tendency (mean, median, and mode), skewness, kurtosis, the Anderson-Darling test, and the Shapiro-Wilk test [46,47]. A distribution was considered normal if it had a two-sided *P* value > .05 for skewness coefficient, kurtosis coefficient, and the Anderson-Darling or Shapiro-Wilk tests [46,47]. The IFCC recommends testing for normal distribution using coefficients of skewness and kurtosis and the Anderson-Darling test [46]. The Shapiro-Wilk test, which has not been evaluated by the IFCC, was also used as it has recently been found to be more powerful than the Anderson-Darling test [47]. For comprehensiveness, z-scores of both skewness and kurtosis ware calculated as [Skewness (or Kurtosis)/SD of Skewness (or Kurtosis)] and z-scores between ±1.96 were considered statistically insignificant [48].

The IFCC-CLSI recommends excluding values from unhealthy individuals and estimating the LRLs from healthy individuals only [20,25]. Thus, LRLs were estimated both in presence and absence of the detected outliers as these outliers might be due to G6PD mutations. As laboratory professionals diverge on the best method to estimate LRL, LRLs and their 95% CIs were estimated by three methods when appropriate: parametric, non-parametric bootstrap based on 500 bootstrap samples and non-parametric rank- based [24,25,41,49]. Lower reference limits were also estimated based on methods of previous studies in order to compare between LRLs based on those methods and those based on method of the IFCC-CLSI. The Harris-Boyd method was used to assess the reliability of gender combined LRLs [50,51]. The combined gender LRLs were considered to be reliable when the larger SD divided by the smaller SD <1.5 and when the normal deviate z value was less than the critical z value of 5. Additionally, the proportion criteria were used for non-normal distributions as it is more accurate than the Harris-Boyd method for such distributions. (Lahti A, 2004), [52] The combined gender LRLs were considered to be reliable when proportion of G6PD values less than the combined LRL did not exceed 4.1% in any gender subgroups [52].

The binomial test was used to compare the observed frequencies of male and female neonates. The two-sample *t*-test assuming equal variances or Mann–Whitney *U* test was used to compare differences between male and female neonates’ continuous variables when appropriate. The two-sample *t*-test assuming unequal variances was used to compare means of this study with those of previous studies. The one-sample Wilcoxon signed rank test was used to compare medians of this study with hypothetical values equal to medians of previous studies. A two-sided *P* value < .05 was considered statistically significant. Data analysis was performed using the RefVal 4.11, IBM SPSS Statistics 20 (Chicago, IL, USA), and OpenEpi 2.2.1 programs.

## Results

**Figure 1:**
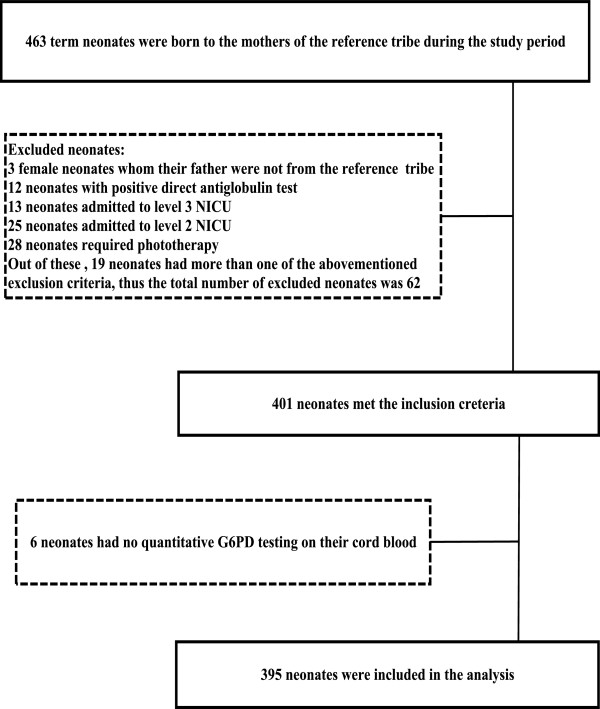
Flow chart describing neonates’ selection.

Figure 1 depicts the process of study selection. A total of 463 term neonates (241 males and 222 females) of the reference tribe were born during the study period. Of these, 62 neonates (13.4%) did not meet the posteriori inclusion criteria (32 males and 30 females). The 2.5th percentile of the G6PD values of the excluded neonates was 9.6 U/g Hb (range: 7.3-19.6). Of the 401 neonates (86.6%) that met the posteriori inclusion criteria (209 males and 192 females), 2 males and 4 females had no quantitative G6PD testing on their cord blood as the reagents were not available at the time of birth. Thus, 395 term neonates with a similar proportion of males (n = 207) and females (n =188) left for analysis (P = .37). Mean (SD) gestational age of male and female neonates were similar [39.6 (1.3) versus 39.6 (1.4) weeks, P = .68). Mean (SD) birth weight of male and female neonates were similar [3315 (460) versus 3243 (442) grams, P = .12).

The combined gender reference sample group had a mean (SD) birth weight of 3281 (452) grams and a mean gestational age of 39.6 (1.3) weeks.

### The outliers

**Figure 2:**
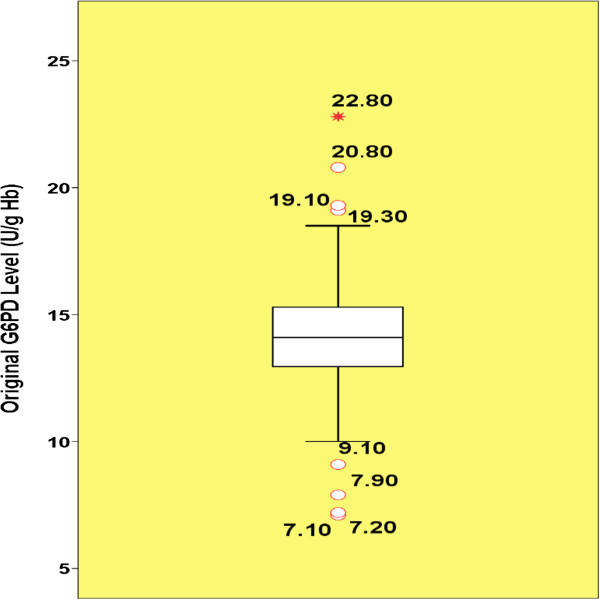
**Tukey’s boxplot for outlier detection in male neonates.** The open circles represent mild outliers which are the G6PD values fall between the Tukey’s inner and outer fences. The star represents extreme outlier which is the G6PD value fall outside the Tukey’s outer fences.

The G6PD values among the males had no significant skewness (0.07). The SPSS syntax indicated that the original data had the lowest skewness. Thus, Tukey’s boxplot was performed on their original values (Figure 2). Both the RefVal and SPSS programs detected exactly the same eight (3.9%) G6PD values as outliers among males (Figure 2).

**Figure 3:**
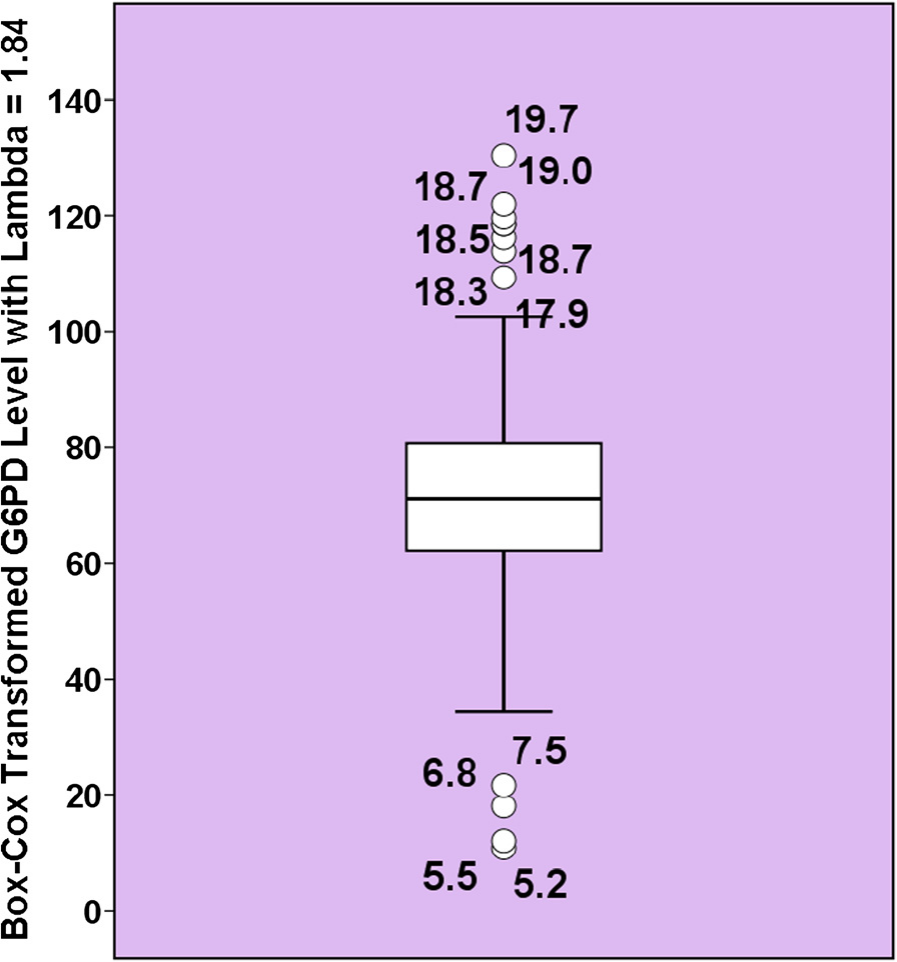
**Tukey's boxplot on box-cox transformed G6PD values of female neonates (method of Horn for outlier detection).** The open circles represent mild outliers which are the G6PD values fall between the Tukey’s inner and outer fences.

The G6PD values among the females had significant negative skewness (-0.93), thus, Tukey’s boxplot was performed on their Box-Cox transformed values (Method of Horn). The SPSS program indicated that a lambda of 1.84 is the best. The Box-Cox transformed G6PD values had a skewness of -0.003. Both the RefVal and SPSS programs detected exactly the same12 (6.4%) G5PD values as outliers among the females (Figure 3).

Some of the outliers were separated by a gap from the rest of the G6PD values (see below). Assuming that the frequency of mutated G6PD alleles among males is 0.039 (number of detect outliers among males/total number of males), the Hardy-Weinberg equation revealed that the expected number of both heterozygous and homozygous females would be 14, nearly the same as number of the detected outliers (P = .87).

### Estimation of LRLs in presence of the outliers

**Figure 4:**
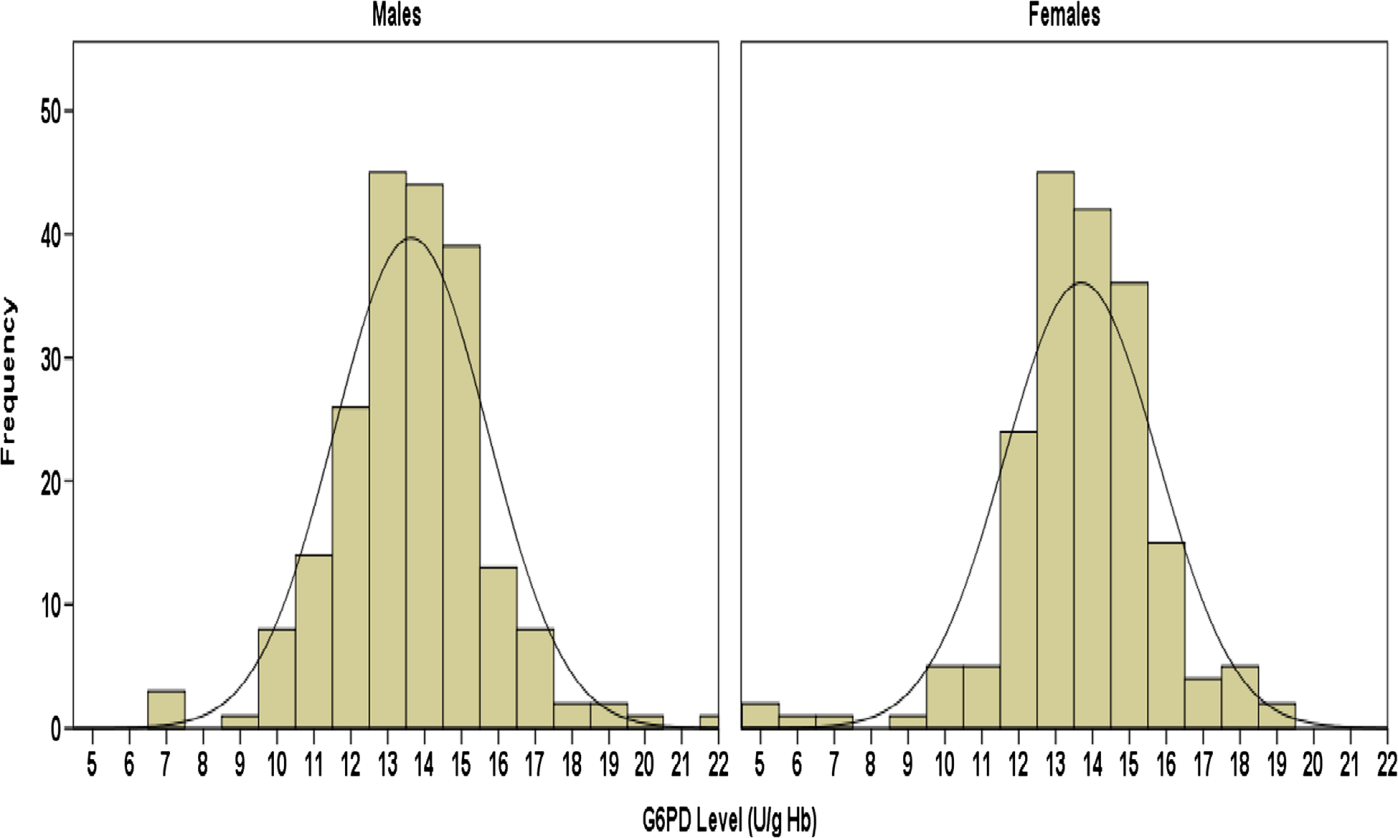
Distributions of quantitative cord G6PD values in male and females in presence of the outliers with superimposed best-fitting normal distribution curves.

**Figure 5:**
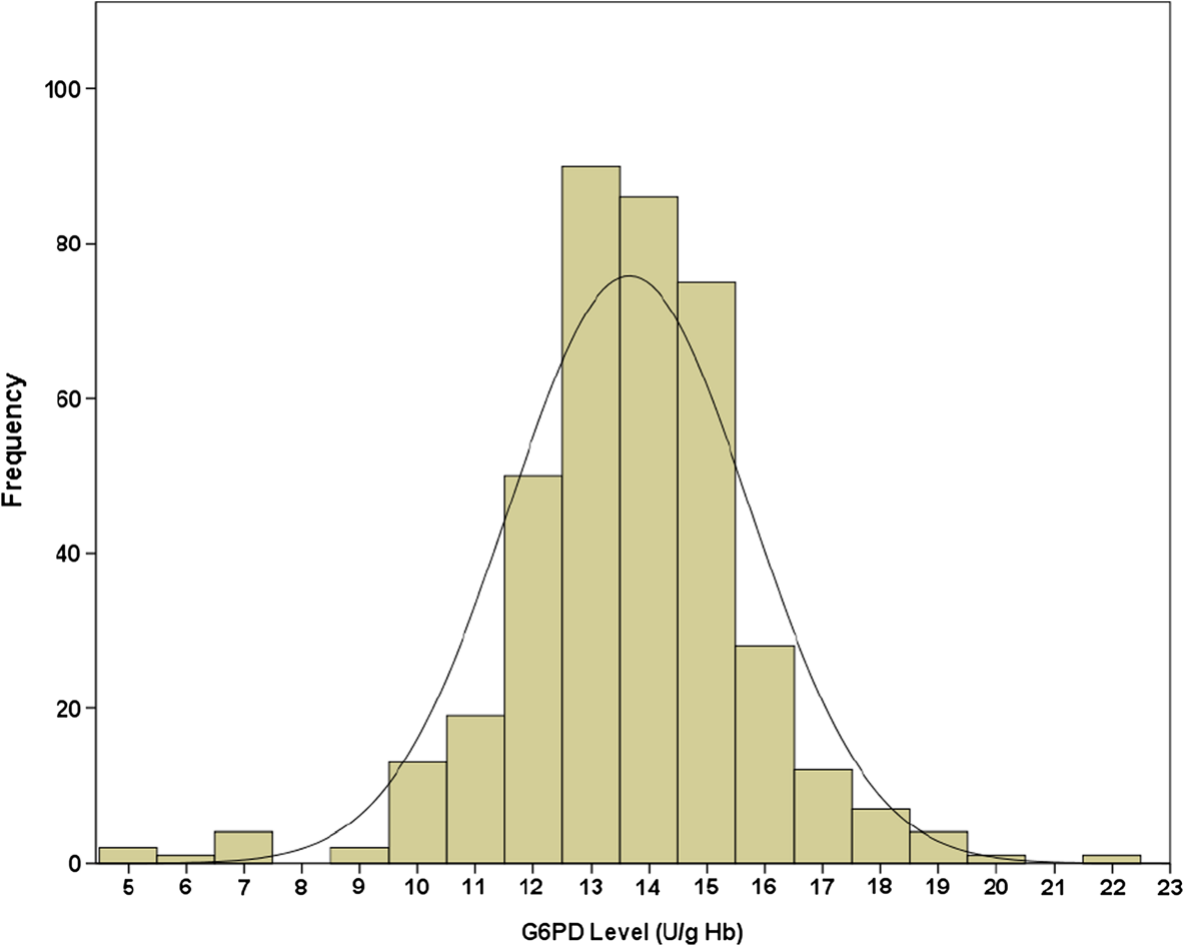
Distributions of quantitative cord G6PD values in the combined gender group in presence of the outliers with superimposed best-fitting normal distribution curve

The histograms (Figures 4 and 5), measures of central tendency, and all statistical tests of normality (Table 1) suggested that the G6PD values of males, females and the combined group exhibited non-normal distributions. The G6PD values of males had one gap in each ends. The gap in lower end bounded by 9.10 and 7.90 U/g Hb and the gap in upper end bounded by 22.80 and 20.80 U/g Hb (Figure 4). The G6PD values of females had one gap in the lower end only which bounded by 9.60 and 7.50 U/g Hb (Figure 4).

**Table 1:** Characteristics of G6PD values and lower reference limits (2.5th percentiles) in presence of outliers

	**Males (n = 207)**	**Females (n = 188)**	**Combined (n = 395)**
Mean (SD)^a^	14.1 (2.1)	14.1 (2.1)	14.1 (2.1)
Median (interquartile range)^a^	14.1 (12.9-15.3)	14.2 (13.2-15.2)	14.1 (13.0-15.3)
Mode^a^	14.7	15.1	13.3
z-score of skewness^b^	0.07/0.17 = 0.41	-0.93/0.18 = 5.2	-0.40/0.12 = -3.3
z-score of kurtosis^b^	2.3/0.34 = 6.8	3.9/0.35 = 11.1	3.0/0.25 = 12.0
Skewness coefficient (*P*-value)	.66	<.001	.002
Kurtosis coefficient (*P*-value)	<.001	<.001	<.001
Anderson-Darling test (*P*-value)	<.001	<.001	<.001
Shapiro-Wilk test (*P*-value)	<.001	<.001	<.001
Two-stage transformation parametric	9.8 (8.8-10.7)	9.4 (7.9-10.6)	Unreliable as transformed distribution is not normal
LRL (95% CI)^a^			
Bootstrap LRL (95% CI)^a^	10.0 (7.3-10.9)	9.0 (5.5-10.9)	10.0 (7.5-10.7)
Non-parametric LRL (95% CI)^a^	10.0 (7.1-10.8)	9.0 (5.2-10.9)	10.0 (7.1-10.7)
Combination tests			
1. Proportion of observations	4/207 (1.9%)	5/188 (2.7%)	
less than the combined LRL			
(proportion criteria)		1.0	
2. Harris-Boyd method		Zero	
Larger SD/Smaller SD		6.4	
Normal deviate z test			
Critical z value of 5			

^a^G6PD activity expressed as Units/gram Hemoglobin.

^b^z-score = Skewness (or Kurtosis)/SD of Skewness (or Kurtosis).

Table 1 depicts that G6PD levels in male and female neonates were similar (P = .50). It depicts the estimated LRLs in presence of the outliers. The RefVal indicated that even the two-stage transformation of the combined group did not yield a normal distribution. As a result, it recommended using the bootstrap method for the combined LRLs. The combined LRLs were reliable as the proportion of G6PD values less than the common LRL (10.0 U/g Hb) did not exceed 4.1% among male or female neonates, the ratio of SDs < 1.5 and the normal deviate z test < the critical z value of 5 (Table 1).

### Estimation of LRLs in absence of the outliers

**Figure 6:**
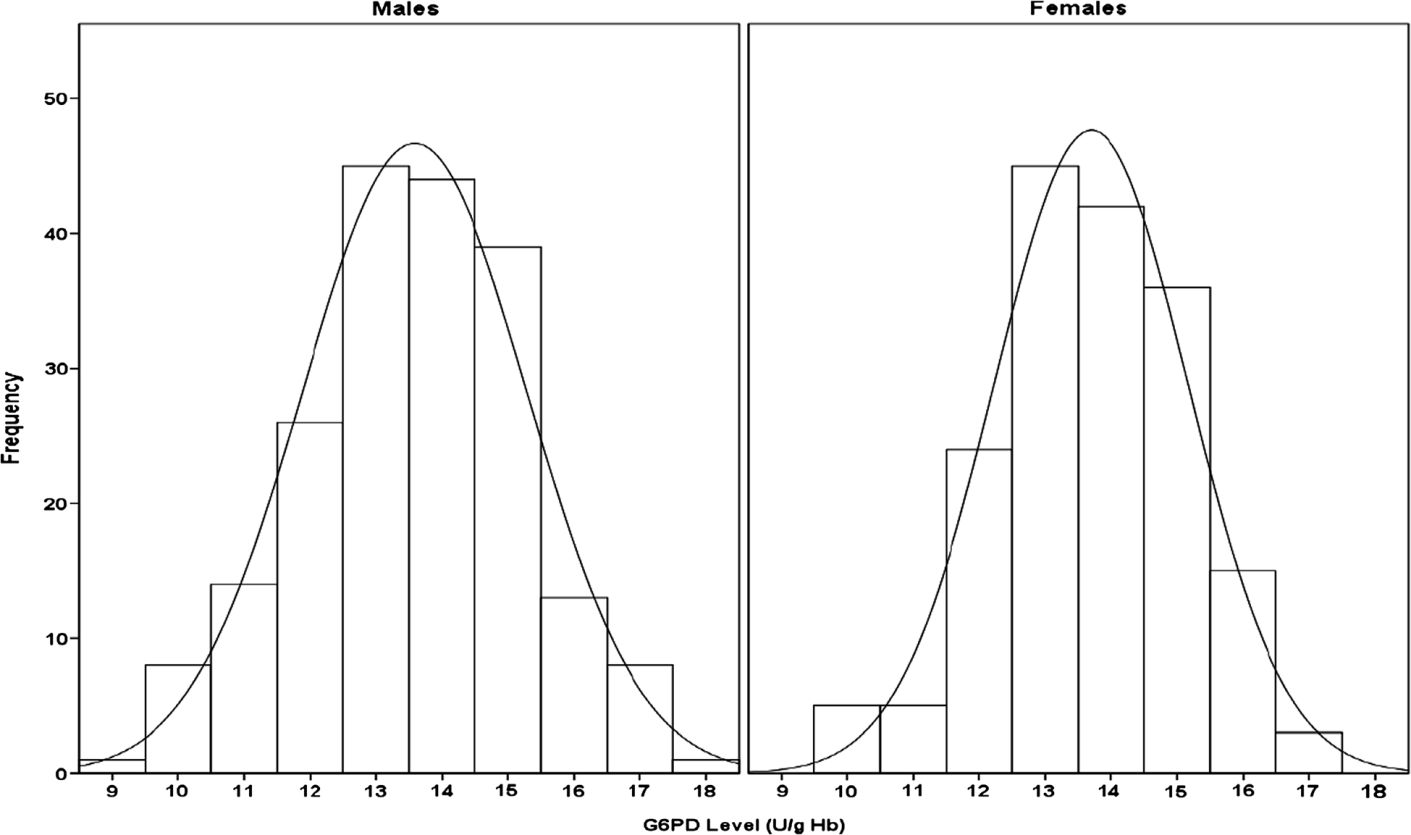
Distributions of quantitative cord G6PD values in male and females in absence of the outliers with superimposed best-fitting normal distribution curves.

**Figure 7:**
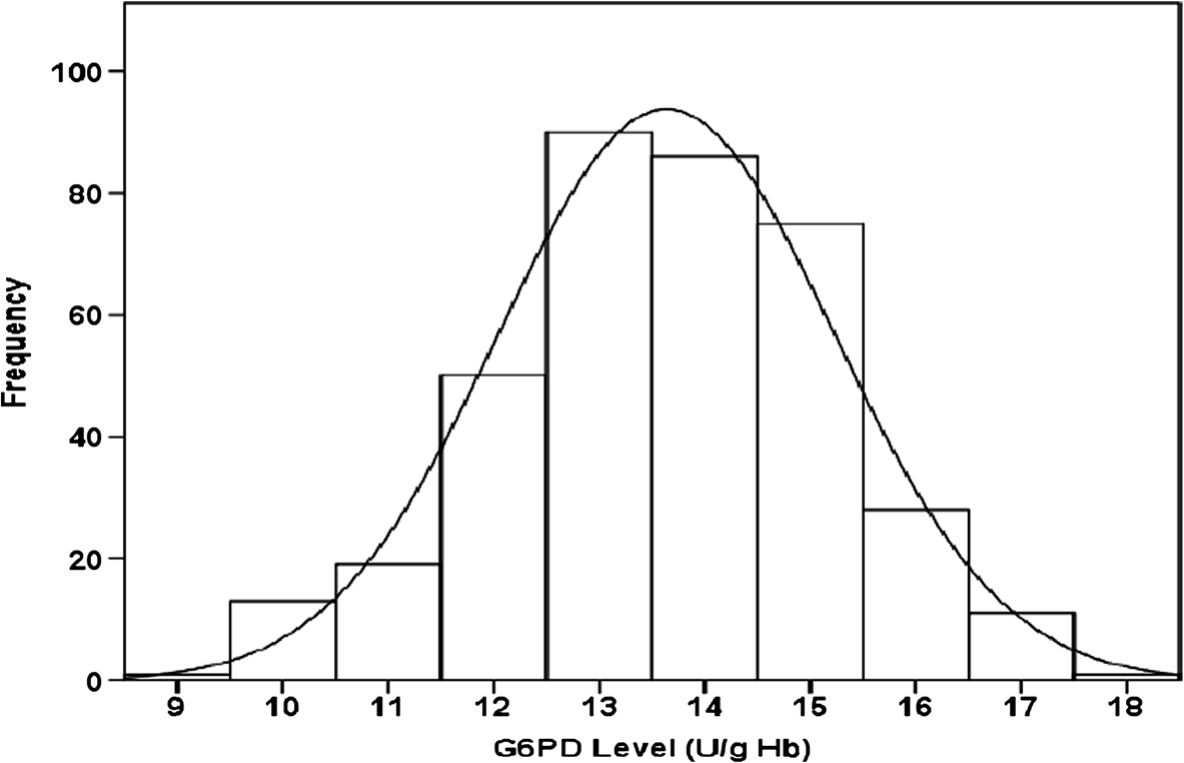
Distributions of quantitative cord G6PD values in the combined gender group in absence of the outliers with superimposed best-fitting normal distribution curve.

After the outliers were excluded, LRLs were estimated from cord blood samples of 375 neonates (199 males and 176 females). The histograms (Figures 6 and 7) and measures of central tendency (Table 2) suggested that the G6PD values of males, females and the combined group all exhibited normal distributions. This was confirmed by the non-statistically significant coefficients of skewness and kurtosis and by the Anderson-Darling and Shapiro-Wilk tests (Table 2). As expected, the observed gaps in presence of the outliers disappeared.

**Table 2:** Characteristics of G6PD values and lower reference limits (2.5th percentiles) in absence of the outliers

	**Males (n = 199)**	**Females (n =176)**	**Combined (n = 375)**
Mean (SD)^a^	14.1 (1.7)	14.1 (1.4)	14.1 (1.6)
Median (interquartile range)^a^	14.1 (13.0-15.2)	14.1 (13.2-15.1)	14.1 (13.1-15.2)
Mode^a^	14.7	15.1	13.3
z-score of skewness^b^	-0.20/0.17 = -1.18	-0.30/0.18 = -1.67	-0.24/0.13 = -1.85
z-score of kurtosis^b^	-0.13/0.34 = -0.38	-0.11/0.37 = -0.28	-0.06/0.25 = 0.24
Skewness coefficient (*P*-value)	.24	.11	.06
Kurtosis coefficient (*P*-value)	.87	.89	.92
Anderson-Darling test (*P*-value)	>.999	>.999	.12
Shapiro-Wilk test (*P*-value)	0.64	0.18	0.14
Parametric LRL (95% CI)^a^	10.8 (10.3–11.3)	11.0 (10.5–11.6)	**11.0 (10.5–11.3)**^**c**^
Bootstrap LRL (95% CI)^a^	10.7 (10.1–11.2)	10.6 (10.2–11.6)	10.7 (10.3–11.1)
Non-parametric LRL (95% CI)^a^	10.7 (10.0–11.2)	10.6 (9.6–11.6)	10.7 (10.2–11.1)
Harris-Boyd method combination test			
Larger SD/Smaller SD		1.2	
Normal deviate z test		Zero	
Critical z value of 5		6.2	

^a^G6PD activity expressed as Units/gram Hemoglobin.

^b^z-score = Skewness (or Kurtosis)/SD of Skewness (or Kurtosis).

^c^The suggested lower reference limit (LRL) and its 95% confidence interval (CI).

Table 2 depicts that G6PD levels in male and female neonates were similar (P = .62). Estimated parametric LRLs had the tightest 95% CI, followed by bootstrap and non-parametric LRLs (Table 2). The combined LRLs were reliable as the ratio of SDs < 1.5 and the normal deviate z test < the critical z value of 5 (Table 2). As a result, the suggested LRL is 11.0 U/g Hb.

Table 3 depicts LRL estimations based on methods of previous studies. They were nearly the same, lower, or higher than those estimations based on statistical analyses of the IFCC-CLSI (Table 3). The observed mean and median G6PD values were nearly the same, lower, or higher than those of 8 previous studies (Table 3).

**Table 3:** Summary of 8 previous studies on the lower reference limit (LRL)/lower decision limit (LDL) of cord G6PD activity and our LRL/LDL estimations based on methods of those studies

**Study**	**Mean (SD)**^**a **^**OR Median**^**a**^	***P-*value**^**b**^	**LRL/LDL**^**a**^	**Our LRL/LDL estimations**^**a **^**based on methods of previous studies**	**Method for LRL/LDL estimation**	**G6PD measurement method**
I. Previous studies with means/medians similar statistically/clinically to those of the present study						
Fok et al. (1985) [8]			10.6	10.8	Chinese neonates born at >30 weeks of gestation.	Cobas Bio,F.Hoffmann, La Roche & Co. (Switzerland)
Male (n = 660)	14.8	<.001^c^			Observed normal G6PD activity separated from abnormal activity in male neonates.	
	14.3 (3.9)	.30				
Female (n = 568)	14.6	<.001^c^			The 3rd percentile of values	
	14.6 (2.9)	.002^c^			after excluding deficient male neonates (< 3.0).	
Ainoon et al. (2003)[10] (n = 976)	14.6 (mean)	SD was not reported^C^	8.7	8.5	Malay and Chinese neonates. Gestational age was not addressed. G6PD deficiency is < 60% of the normal mean level.	Randox Laboratories, Ltd.
Riskin et al. (2012) [15]	14.7 (2.0)	<.001^c^			Jew (Sephardic, Ashkenazi, Ethiopian), Arab (Muslim, Druze, Christian), and Caucasus preterm and term neonates.	Sentinel Diagnosticskit (Italy)
(n = 2269 term neonates)						
Male			>7.0	8.5	G6PD deficiency is < 60% of the normal mean level. [70] Gender distribution. Hardy-Weinberg equation.	
Female			>10.0	9.6		
II. Previous studies with means lower than those of the present study						
Boo et al. (1994) [7]			>4.1	9.9	Normal Malay, Chinese, and Indian neonates born at 37 weeks of gestation with	Manually according to the standardized
Male (n = 135)	8.3 (2.2)	<.001			G6PD level 4.1 U/g Hb or negative fluorescence spot test.	WHO method for G6PD assay of the hemolysate.
	95% CI:7.9-8.6	<.001				
Female (n = 127)	8.5 (2.1)					
	95% CI:8.2-8.9				Mean-2SD	
Azma et al. (2010) [11] (n = 94)	12.4 (2.3)	<.001	10.2	12.0	Normal term Malay neonates with negative fluorescence spot test. 68% reference interval (mean-1SD)	OSMMR-D (R&D Diagnostics Ltd., Greece)
III. Previous studies with mean/median higher than those of the present study						
Reclos et al.(2003) [9]					Uneventful pregnancies and normal full-term deliveries.	OSMMR200 0 (R&D
Greek Male (n = 505)	20.8 (1.6)	<.001	12.5	8.5	G6PD deficiency is < 60% of the normal mean level.[70]	Diagnostics Ltd., Greece)
Greek Female (n = 551)	19.5 (2.0)	<.001	11.7	8.5		
Albanian Male (n = 444)	21.6 (2.0)	<.001	13.0			
Albanian Female (n = 363)	21.0 (2.6)	<.001	12.6			
Kaplan et al. (2005) [13]					Healthy term and near-term African American neonates.	Technicon RA 1000 analyzer (Bayer Diag.,NY)
Male (436)	21.8 (2.9)	<.001	14.5	9.1	Observed normal G6PD activity separated from abnormal activity in male neonates.	
Algur et al. (2012) [16]					Sephardic Jew born at 36 weeks of gestation.	Sentinel Diagnostics kit (Italy)
Male (n = 1256)	18.8	<.001	9.0	9.1	Males: observed normal G6PD activity separated from abnormal activity.	
Female (n = 1153)	18.4	<.001	9.5	7.1^d^	Probable normal females: > 50% of the normal male median level.	

^a^G6PD activity expressed as Units/gram Hemoglobin (U/g Hb). Some of the original values were rounded to one decimal.

^b^*P*-value for the two-sample *t*-test that was used to compare our means with those of previous studies and the one-sample Wilcoxon signed rank test that was used to compare our medians with those of previous study.

^c^Not clinically significant.

^d^Our estimated LRL would be 9.6 U/g Hb based on the observed gap in G6PD values of females.

## Discussion

The present study estimated the LRLs of quantitative cord G6PD using standard statistical methods endorsed by the IFCC-CLSI for establishing RIs [24,25]. The study used a homogenous reference sample group of healthy term neonates expected to have a low prevalence of G6PD deficiency. The results showed that 11.0 U/g Hb is a reliable combined parametric LRL for both genders. This value may help to identify G6PD-deficient heterozygous female neonates [28,53]. This LRL can be transferred to other laboratories using a similar G6PD measurement method, satisfying the conditions for transference validation.

Reference limits are the cornerstones of interpretation of quantitative cord G6PD activity measurements. Standardization of statistical analysis for RI estimation is critical and is enabled by the IFCC-CLSI’s endorsement of a standard statistical analysis for RI estimation [24,25]. Yet, the present study is the first to implement this standard method of statistical analysis to estimate one-sided RIs of quantitative cord G6PD. It demonstrated clearly that different methods are yielding different LRL estimations as most of our estimations based on methods of previous studies were inconsistent with our estimations based on method of the IFCC-CLSI (Table 3). Thus, standardized statistical analysis and reporting of studies on LRL estimations should be encouraged.

Our observed gaps in G6PD values of males confirmed the findings of others [8,13,16,54]. Apparently, presence of these gaps depends on type of G6PD mutation and age of RBCs [55]. An old Italian study found that 98-100% of RBC of 115 normal schoolboys (G6PD-B) were G6PD (+), 98-100% of RBCs of 45 schoolboys bearing G6PD-Meditrianian were G6PD (-), and six schoolboys bearing G6PD-Seattle-like had a mosaic population of G6PD (+) and (-) RBCs [54]. For that reason, gaps were observed between G6PD values of G6PD-Meditrianian and normal boys while no gap was observed between G6PD-Seattle-like and normal boys [54]. Having said that, our observed gaps developed as their outermost borders and afterward values might be due to pre-analytic errors.

Our observed gap in the cord G6PD values of females has not been reported before. It is rather contradicting that G6PD values of females are continuum as heterozygotes may express a spectrum of values; normal, intermediate, and deficient [16,56]. The intermediate values will bridge the gap between normal and deficient values. That is why it is difficult to diagnosis heterozygotes without a family history or molecular testing [56-59]. Thus, this observed gap developed by chance or because their outermost borders and afterward values might be due to pre-analytic errors. However, excessive skewing of X-chromosome inactivation (allele ratios 3:1) in heterozygotes might account partly for our observed gap in G6PD values of females [60]. The excessive skewing has been reported to be in favor of RBCs bearing normal G6PD gene [54]. As a result, G6PD values for heterozygotes would be within RI of non-G6PD deficient females. On other hand, the skewing could be in favor of abnormal RBCs, and then G6PD values for heterozygotes would be within RI of homozygous G6PD-deficient females [60,61]. Reported incidence of excessive skewing of X-chromosome in cord blood of healthy, term, female neonates is 9- 24% [62,63]. Nevertheless, as the observed gap could be due to pre-analytic errors or chance and has no plausible explanation, further confirmatory and exploratory studies are required before firm conclusions can be drawn.

Comprehensive normal distribution testing in the present study showed that the distribution of quantitative cord G6PD exhibited a normal distribution only in absence of the outliers. Previous studies that have run limited normal distribution testing diverge on the normal distribution of G6PD values. The G6PD values exhibited a normal distribution in the study by Boo et al. but a non-normal distribution in the study by Fok et al. [7,8]. The fact that only full-term neonates were included in both the present study and that by Boo et al. might explain the normal distribution in those populations, while the inclusion of both preterm and term neonates in the study by Fok et al. might account for the non-normal distribution, as G6PD activity has been shown to vary with gestational age (Table 3) [15,36,37]. Some researchers based their LRL and LDL estimations on the mean without addressing normal distribution testing [13]. Taken together, these observations emphasize the importance of conducting comprehensive normal distribution testing or estimating non-parametric RIs that do not make any assumptions about the data distribution [26].

Outliers are known to skew statistical tests based on sample means and variances, making it strongly advisable to detect outliers before performing these tests. Thus, it was not surprising that presence of these outliers widened the 95% CIs and distorted the homogeneity and the normal distributions, particularly for the combined gender group where even the two-stage transformation did not yield a normal distribution. The IFCC-CLSI emphasizes retaining outliers unless they are due to human or laboratory errors or representing unhealthy individuals. Retrospective nature of study precludes ascertaining whether or not these outliers were due to human or laboratory errors. However, there were three evidences suggesting that the outliers in the present study might be due to G6PD mutations. First, the frequencies of these outliers were in Hardy-Weinberg proportions. Second, presence of gaps between some of these outliers and the rest of presumed normal G6PD values. Third, a recent study from Jeddah area of western Saudi Arabia that found all the 20 female neonates with a cord G6PD value 6.6 U/g Hb subjected to molecular testing were G6PD- deficient [64]. However, the Jeddah study did not subject female neonates with a cord G6PD value > 6.6 U/g Hb to molecular testing [64].

We are suggesting using estimated LRLs in absence of the outliers for three reasons. First, the outliers of the present study might be due to pre-analytic errors. Second, they might be due to G6PD mutations. Third, all estimated LRLs were higher in absence than in presence of the outliers. Increasing cut-off point of LRLs will increase their sensitivity at the expense of their specificity. This is quite acceptable as mislabeling G6PD-deficient neonates as normal is intolerable whereas mislabeling G6PD-normal neonates as deficient is tolerable [65]. The parametric method is preferable to bootstrap and non-parametric methods for normal distributions [49]. This study showed that parametric LRLs had tight 95% CIs. The reference sample group in the present study satisfied the assumptions of the Harris-Boyd method as the G6PD values exhibited a normal distribution and the proportions of males and females were similar [50]. The Harris-Boyd method showed that the LRL of the combined genders is reliable. The standard statistical tests, the two-sample *t*-test assuming equal variances and Mann–Whitney *U* test also showed that G6PD levels were similar in male and female neonates. This is logic and consistent with the fact that the LRL was estimated from non-G6PD deficient neonates, as normal males and females should have the same G6PD values [58]. Consequently, we suggested the parametric LRL of the combined gender group (11.0 U/g Hg) as the LRL for cord G6PD of term neonates. Future studies on the LRLs for preterm neonates using the IFCC-CLSI are warranted as G6PD activity has been shown to vary across gestational age categories [15,36,37].

Some laboratory professionals have advocated estimating the 5.0th percentile as the LRL when a one-sided RI is assumed [66]. We elected the 2.5th percentile as the LRL in compliance with the IFCC-CLSI guidelines. However, the 5.0th percentiles of the G6PD values in this study were similar to the 2.5th percentiles (data not shown).

The IFCC-CLSI allow transferal of a reference limit from one laboratory to another when pre-analytical factors and testing methods are comparable in both laboratories and when one of their 3 validation methods is satisfied [25]. One of these validation methods is the N-20 reference sample group method, in which 20 samples from carefully assessed reference individuals are tested. When no more than 2 of these 20 samples fall outside the transferred reference limit, it is statistically valid to accept the transferred reference limit. Various commercial G6PD kits are available (Table 3), but as few studies have addressed their comparability, [65] future studies using the CLSI guidelines for methodological comparison are warranted [67].

Observed mean and median G6PD values were statistically significantly different from those reported in previous studies. The discrepancy could be due to differences in the timing of these studies, the ethnicities of the reference sample groups, gestational ages, G6PD kits and their reagents, and statistical strategies for LRL estimation (Table 3). However, not all of these differences are clinically significant, as trivial differences were sometimes statistically significant due to large sample sizes (Table 3).

Controversy exists as to whether G6PD activity should be expressed as U/g Hb or per number of RBCs. The WHO and some researchers have endorsed expressing the G6PD activity per number of RBCs to per U/g Hb, as the hemoglobin content may fluctuate independently of the G6PD activity, particularly when RBCs are hypochromic [6,12]. Other researchers have compared the two expressions using neonatal peripheral blood and found that they are perfectly correlated [35]. However, the correlation between the two expressions of cord G6PD activity still needs to be determined.

The present study has four limitations that should be noted. First, retrospective nature of the study precluded assuring that the grandmothers were form the reference tribe as their names were not printed in the birth notices. Nevertheless, we do not assume that prevalence of neonates of grandmothers who were not from the reference tribe was high enough to alter prevalence of G6PD deficiency in the reference tribe significantly. The intermarriages between the reference tribe and other tribes are not a familiar tradition. The intermarriages between Bedouin descents and urban descents rarely occurred. For instance, in the present study, 3 out of 222 (1.4%) female neonates were product of intermarriages between the reference tribe and other tribes that all were Bedouin descents. Second, reported LRLs were not cross-referenced with molecular testing, so a future study using molecular testing to verify these LRLs is warranted. Third, the G6PD activity was only expressed as U/g Hb, precluding comparison with other studies that expressed G6PD activity per number of RBCs [12]. Fourth, methods of Horn and Harris-Boyd have some limitations that have been addressed elsewhere; yet, these are the best available methods for assessing outliers and common LRL, respectively [51,68,69]. However, even the standard statistical tests, the two-sample *t*-test assuming equal variances and Mann–Whitney *U* test also showed that G6PD levels were similar in male and female neonates.

## Conclusions

The present study estimated the LRLs of cord G6PD activity using the standard method of statistical analysis endorsed by the IFCC-CLSI. We believe that we have included healthy neonates as we used stringent inclusion criteria and we included a reference group that has a low prevalence of G6PD deficiency. The results showed that the LRL of cord G6PD activity for full-term neonates of both genders is 11.0 U/g Hb and that this value is transferable to other settings when the IFCC-CLSI requirements of transference are satisfied. Further studies are warranted on LRLs for preterm neonates using the statistical standards of the IFCC-CLSI, cross-referencing the LRL with molecular testing, comparing methods of expressing cord G6PD activity, and comparing various commercial kits. Standardized statistical analysis and reporting of studies on RI estimation should be encouraged.

## Abbreviations

KAH: King Abdulaziz Hospital; G6PD: Glucose-6-phosphate dehydrogenase; LRL: Lower reference limit; LDL: Lower decision limit; RI: Reference interval; IFCC: International federation of clinical chemistry; CLSI: Clinical laboratory standard institute; WHO: World Health Organization; u/gHb: Units/gram hemoglobin; RBCs: Red blood cells; FST: Fluorescence spot test; °C: Degree celsius; EDTA: Ethylenediaminetetraacetic acid; NADP: Nicotinamide adenine dinucleotide phosphate; IQR: Interquartile range; 95% CI: 95% Confidence interval.

## Competing interests

We do not have any conflicts of interest to disclose.

## Authors’ contributions

SYA conceptualized and designed the study, double checked and cleaned data, performed the statistical analysis, drafted the initial manuscript, and approved the final manuscript as submitted. He had full access to all the data in the study and takes responsibility for the integrity of the data and the accuracy of the data analysis. ASS contributed substantially to the designing the study, data collection, interpretation of this work, reviewed and revised, and approved the final manuscript as submitted. FLD contributed substantially to the designing the study, data collection, interpretation of this work, reviewed and revised, and approved the final manuscript as submitted. AAS contributed substantially to the designing the study, data collection, interpretation of this work, reviewed and revised, and approved the final manuscript as submitted. All authors read and approved the final manuscript.

## Pre-publication history

The pre-publication history for this paper can be accessed here:

http://www.biomedcentral.com/1471-2431/13/137/prepub
